# Structure of an RNA helix with pyrimidine mismatches and cross-strand stacking

**DOI:** 10.1107/S2053230X19012172

**Published:** 2019-09-24

**Authors:** Eric J. Montemayor, Johanna M. Virta, Lauren D. Hagler, Steven C. Zimmerman, Samuel E. Butcher

**Affiliations:** a Department of Biochemistry, University of Wisconsin–Madison, Madison, WI 53706, USA; b Department of Chemistry, University of Illinois Urbana–Champaign, Urbana, IL 61801, USA

**Keywords:** RNA, myotonic dystrophy type 1, pyrimidine mismatches

## Abstract

The structure of a 22-base-pair RNA helix with mismatched pyrimidine base pairs is reported. The helix contains two symmetry-related CUG sequences: a triplet-repeat motif implicated in myotonic dystrophy type 1. The CUG repeat contains a U–U mismatch sandwiched between Watson–Crick pairs. Additionally, the center of the helix contains a dimerized UUCG motif with tandem pyrimidine (U–C/C–U) mismatches flanked by U–G wobble pairs. This region of the structure is significantly different from previously observed structures that share the same sequence and neighboring base pairs. The tandem pyrimidine mismatches are unusual and display sheared, cross-strand stacking geometries that locally constrict the helical width, a type of stacking previously associated with purines in internal loops. Thus, pyrimidine-rich regions of RNA have a high degree of structural diversity.

## Abstract 2

The structure of a 22-base-pair RNA helix with mismatched pyrimidine base pairs is reported. The pyrimidine mismatches are unusual and display sheared, cross-strand stacking geometries that locally constrict the helical width.

## 1. Introduction   

Cellular transcriptomes are large with myriad important biological functions. However, only a few percent of the structural coordinates in the Worldwide Protein Data Bank correspond to RNA, and some of these are redundant. At the time of writing, there are 1213 unique RNA-containing structures at a resolution of 3 Å or higher in the RNA 3D Motif Atlas (Petrov *et al.*, 2013 ▸). From these data it is apparent that RNA molecules are structurally diverse. Whereas many structural motifs have been described for RNA (Butcher & Pyle, 2011 ▸), it is likely that new motifs will be discovered as more structures are solved.

Myotonic dystrophy type 1 (DM1) is a heritable disease caused by the expansion of genomically encoded CUG repeats in the 3′ untranslated region of the dystrophia myotonica protein kinase (DMPK) mRNA (Mirkin, 2007 ▸). The CUG repeats are thought to form hairpin stem-loop structures that sequester the splicing factor muscleblind-like protein 1 (MBNL1), resulting in splicing defects (Miller *et al.*, 2000 ▸). Crystal structures of RNAs containing CUG repeats have previously been determined (Coonrod *et al.*, 2012 ▸; Kiliszek *et al.*, 2009 ▸; Kumar *et al.*, 2011 ▸; Mooers *et al.*, 2005 ▸). In these structures, the CUG repeats are composed of C–G base pairs that sandwich U–U mismatches. Previous structures have shown that the U–U mismatches can adopt heterogeneous structures, with either zero, one or two hydrogen bonds (Coonrod *et al.*, 2012 ▸; Kiliszek *et al.*, 2009 ▸; Kumar *et al.*, 2011 ▸; Mooers *et al.*, 2005 ▸).

The UUCG tetraloop is one of the most stable and commonly occurring RNA loop sequences (Cheong *et al.*, 1990 ▸). Structures of the UUCG tetraloop have been determined (Allain & Varani, 1995 ▸; Ennifar *et al.*, 2000 ▸; Nichols *et al.*, 2018 ▸; Nozinovic *et al.*, 2010 ▸). It has previously been observed that during crystallization RNA hairpins containing UUCG tetraloops can dimerize into double helices in which the UUCG sequence forms non-Watson–Crick base pairs (Berger *et al.*, 2019 ▸; Cruse *et al.*, 1994 ▸; Holbrook *et al.*, 1991 ▸). The two previous crystal structures of dimerized UUCG sequences contain U–G wobble pairs flanking mismatched U–C pairs that are bridged by an intervening water molecule.

**Figure 1:**
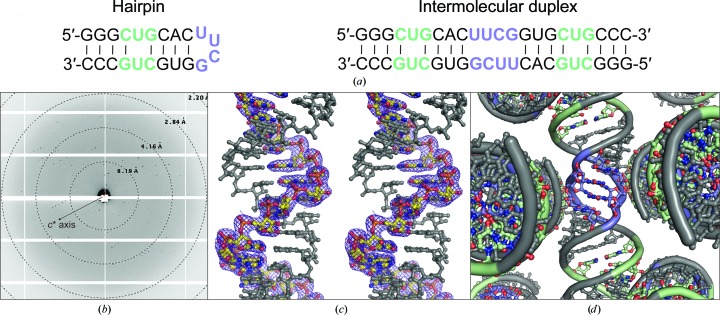
Sequence and structure of a dimerized RNA with an unusual UUCG duplex motif. (*a*) Sequence of the anticipated hairpin structure and the observed dimerized structure. The CUG repeat region is colored green and the expected UUCG tetraloop is colored blue. (*b*) Example diffraction data collected from a single crystal. The oscillation range in the depicted image corresponds to a total of 2°, summed together from ten adjacent 0.2° oscillation images. The c* axis is indicated with an arrow. (*c*) Cross-eyed stereo image of the final electron-density map. One chain in the asymmetric unit is colored yellow and is related to the other chain (gray) by crystallographic symmetry, thus generating an RNA duplex *in crystallo*. The depicted map is of the form 2*mF*_o_ − *DF*_c_, is unfilled for missing reflections and is contoured at 1 r.m.s.d. Density is only shown within 2 Å of the modeled chain within the crystallographic asymmetric unit. (*d*) Crystal packing of the RNA duplex in the vicinity of the dimerized UUCG motif. The coloring is the same as in (*a*).

Here, we report the crystal structure of an RNA that contains a CUG repeat and a UUCG sequence. The RNA was designed to form a hairpin with an isolated CUG repeat [Fig. 1 ▸(*a*)] to provide a platform for analyzing compounds designed to target the CUG repeat sequence (Arambula *et al.*, 2009 ▸). Instead, the RNA crystallized into a duplex in which the two CUG repeats are related by twofold symmetry and form a U–U mismatch flanked by C–G pairs. In the CUG repeat, the U–U base pair has two hydrogen bonds. The dimerized UUCG sequence displays novel cross-strand stacking of pyrimidine pairs, with inter-strand hydrogen bonds between the uracil nucleobase on one strand and the uracil ribose 2′ O atom of the opposite strand.

## 2. Materials and methods   

### 2.1. RNA production   

A putative RNA hairpin (5′-GGGCUGCACUUCGGUGCUGCCC-3′) was purchased from Integrated DNA Technologies. The synthesized RNA was resuspended in anion-exchange buffer (300 m*M* NaCl, 20 m*M* potassium phosphate pH 6.5, 1 m*M* EDTA, 1 m*M* sodium azide) and immobilized on a 1 ml HiTrap Q column (GE Healthcare). The column was washed with ten volumes of buffer prior to step elution in anion-exchange buffer supplemented with 2 *M* NaCl. The resulting eluate was concentrated using centrifugal filters with a 3 kDa cutoff (Amicon) and then iteratively diluted tenfold and reconcentrated three times using a buffer containing only 20 m*M* deuterated bis-Tris pH 6.5. The RNA was then concentrated to 150 µ*M*. A small aliquot of this RNA was resolved on an analytical nondenaturing polyacrylamide gel, which showed a trace amount (∼5%) of RNA migrating as a slower species that is presumed to be an intermolecular dimer (data not shown).

The RNA was subsequently concentrated to approximately 1.5 m*M* (∼10 mg ml^−1^) prior to monitoring its association with the compound ‘JFA’ (Arambula *et al.*, 2009 ▸) via ^1^H NMR (data not shown). For this process, the compound JFA was in 100% DMSO and was added stepwise to a final approximate twofold stoichiometric excess, resulting in a 300 µl sample containing approximately 800 µ*M* RNA, 1600 µ*M* JFA, 5% DMSO, 5% D_2_O and 20 m*M* deuterated bis-Tris pH 6.5%. The RNA with ‘JFA’ was finally concentrated using 3 kDa cutoff centrifugal filters (Amicon) to a volume of approximately 100 µl without additional treatment before crystallization screening.

### 2.2. Crystallization, structure determination and refinement   

High-throughput crystallization screening was performed by sitting-drop vapor diffusion in 96-well plates at 4°C using 0.2 µl RNA solution, 0.2 µl crystallization reagent and a reservoir volume of 50 µl with a Mosquito crystallization robot (TTP Labtech). After a few weeks, several small crystals (∼10 × 50 µm) were obtained using a crystallization reagent consisting of 0.1 *M* HEPES pH 7.4, 20% PEG 3350, 20% glycerol, 10% MPD. Crystals were harvested with 100 µm LD MicroLoops (MiTeGen) and vitrified via rapid immersion in liquid nitrogen.

Diffraction data were collected on NE-CAT beamline 24-ID-E at the Advanced Photon Source using an MD2 diffractometer and an EIGER 16M detector. All scientific software was managed though a local SBGrid client (Morin *et al.*, 2013 ▸). The data were integrated using *XDS* (Kabsch, 2010 ▸). Initial point-group estimation and scaling were performed in *POINTLESS* (Evans, 2011 ▸) and *AIMLESS* (Evans & Murshudov, 2013 ▸), respectively. *Xtriage* (Adams *et al.*, 2010 ▸) was used to assay potential twinning in the diffraction data after identification of the correct space group (see below).

Initial phases were determined by molecular replacement using *Phaser* (McCoy *et al.*, 2007 ▸) with ideal A-form duplex RNA as the initial search model. Molecular replacement was attempted in all possible space groups within the *P*4 point group. A single solution in space group *P*4_1_2_1_2 yielded an initial map of sufficient quality to determine that the RNA was in the form of an intermolecular dimer rather than the anticipated hairpin structure. Manual model building was performed in *Coot* (Emsley *et al.*, 2010 ▸) and subsequent automated refinement and model validation in *PHENIX* (Afonine *et al.*, 2012 ▸) and *REFMAC* (Murshudov *et al.*, 2011 ▸; Winn *et al.*, 2011 ▸). All figures were prepared with *PyMOL* (http://www.pymol.org). Coordinates and structure factors have been deposited in the Protein Data Bank under accession code 6e7l; diffraction images are available from the SBGrid Data Bank at https://doi.org/10.15785/SBGRID/712.

## 3. Results   

The 22-nucleotide RNA strand contains two CUG repeats and a UUCG sequence, and is capable of forming a hairpin or a duplex conformation [Fig. 1 ▸(*a*)]. The crystals diffracted X-rays to 2.59 Å resolution [Fig. 1 ▸(*b*) and Table 1 ▸]. The electron density was well resolved for the entire RNA, which formed an intermolecular duplex in the crystal with the two strands related by twofold crystallographic symmetry; thus, only one stand of the duplex is present in the crystallographic asymmetric unit [Fig. 1 ▸(*c*)]. For the purposes of discussion, we give one strand in the duplex the numbering 1–22 and the other 1′–22′. The RNA adopts an A-form geometry for all nucleotides except the UUCG sequence regions, which are involved in crystal contacts [Figs. 1 ▸(*d*) and 2 ▸(*a*)].

**Figure 2:**
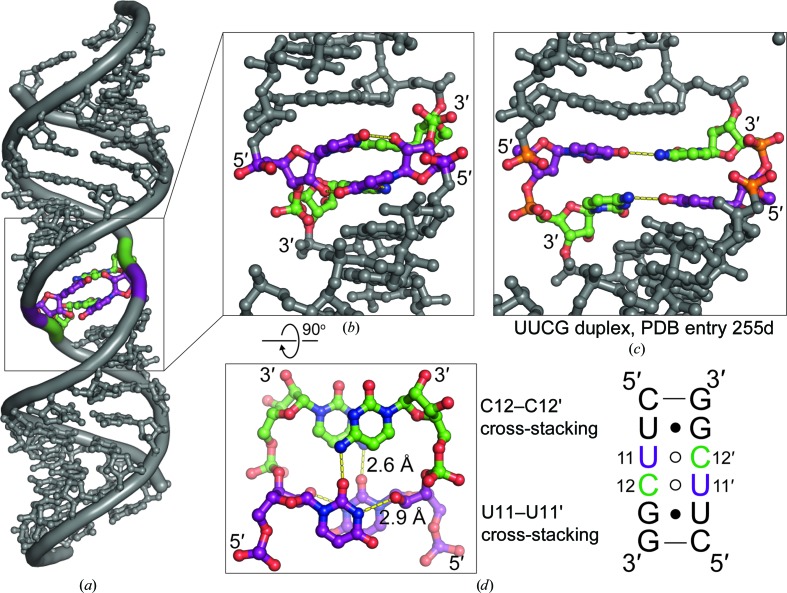
Cross-strand stacking in an intermolecular UUCG–UUCG motif. In the schematic, Watson–Crick base pairing is depicted by straight lines, G–U wobbles by solid black circles and non-Watson–Crick pairing by open circles. (*a*) Overview of the observed duplex. (*b*) The UUCG dimerization interface comprises a compressed helical structure with an approximate diameter of 12 Å owing to cross-stand base stacking. (*c*) A previously observed UUCG dimerization interface lacked cross-strand stacking and was wider, with an approximate diameter of 18 Å. (*d*) In addition to cross-strand stacking, dimerization of the UUCG motif utilizes twofold-symmetric hydrogen bonding between the 2′ OH and the N3 of neighboring uridine nucleotides (U11 and U11′ in the figure and the adjacent schematic) and hydrogen bonding between N4 of cytidine and O2 of uridine across the duplex (U11 and C12′ and also U11′ and C12).

All ribose sugar puckers are C3′-*endo*, with the exception of U11 and U11′, which are C2′-*endo*. The UUCG region forms an unusual structure, with two U–C base pairs that are cross-strand stacked [Fig. 2 ▸(*b*)]. The U–C base pairs form a hydrogen bond between the uracil O2 and the cytosine N3 amino group. An additional inter-strand hydrogen bond is formed between the uracil N3 and the uracil ribose O2′. This conformation is significantly different from previous structures of the same sequence, which lacked cross-strand stacking (Berger *et al.*, 2019 ▸; Cruse *et al.*, 1994 ▸; Holbrook *et al.*, 1991 ▸) [Fig. 2 ▸(*c*)]. The cross-strand stacked U–C base pairs are flanked by U–G wobble pairs. The U–G wobble-pair region is involved in helical packing within the crystal lattice [Fig. 1 ▸(*d*)], mediated by minor-groove interactions that are stabilized by inter-helical hydrogen bonds involving 2′ hydroxyl groups, similar to ‘ribose-zipper’ interactions (Tamura & Holbrook, 2002 ▸).

**Figure 3:**
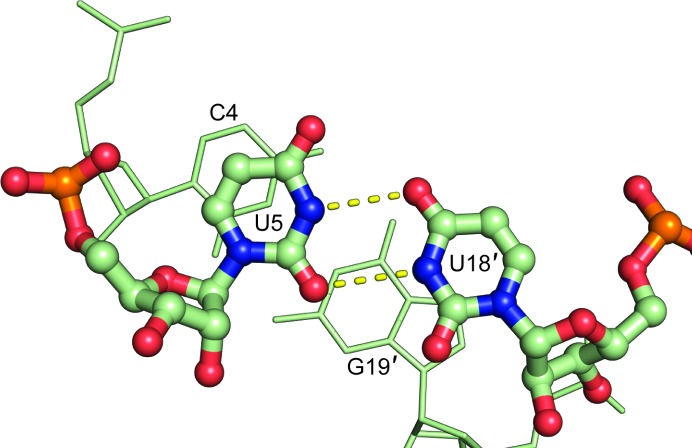
(*a*) The U–U base pair in the CUG repeat regions forms a ‘type V’ pair. (*b*) The U5–U18′ and U5′–U18 pairs are identical owing to twofold symmetry.

The two CUG regions are symmetry-related, with identical structures. The CUG repeat structure is composed of a Watson–Crick C–G pair, a noncanonical U–U pair with two hydrogen bonds and a Watson–Crick G–C pair. The U–U base pair has hydrogen bonds between the imino N atoms and the O2 and O4 atoms (Fig. 3 ▸). This type of U–U base pair has previously been termed a ‘type V’ pair (Fig. 3 ▸; Coonrod *et al.*, 2012 ▸).

## 4. Discussion   

(CUG)_*N*_ repeats in RNA (where *N* is the number of repeats) form helices with U–U mismatches that display heterogeneous base-pairing patterns (Coonrod *et al.*, 2012 ▸; Kiliszek *et al.*, 2009 ▸; Kumar *et al.*, 2011 ▸; Mooers *et al.*, 2005 ▸). The base-paired 5′-CUG-3′ sequences in the structure reported here are symmetry-related and form a ‘type V’ base pair (Fig. 3 ▸), which has been observed previously by crystallography (Kumar *et al.*, 2011 ▸) and NMR (Parkesh *et al.*, 2011 ▸). The CUG repeat is predominately A-form, with a small degree of cross-strand overlap that places the central uridine within van der Waals radius of the guanosine on the opposite strand. This slight degree of cross-strand stacking has been noted previously in the structure of (CUG)_6_ (Mooers *et al.*, 2005 ▸). The geometry of the U–U wobble places the O2 and O4 ketone O atoms in close proximity. While we do not observe associated cations in this structure, the close approach of ketone O atoms in G–U wobble pairs is known to create a cation-binding site, which can be utilized for phasing (Keel *et al.*, 2007 ▸).

Cross-strand stacking in RNA tertiary structure typically involves purines (Chen *et al.*, 2005 ▸; Correll *et al.*, 1997 ▸; Gautheret *et al.*, 1994 ▸; Lee *et al.*, 2006 ▸; SantaLucia *et al.*, 1990 ▸). To our knowledge, the dimerized UUCG structure reported here is a very rare example of a pyrimidine-only interaction with extensive cross-strand stacking. One other known example of cross-strand pyrimidine stacking in RNA occurs in the low-pH structure of the i-motif, which involves intercalated and cross-strand stacked cytidines (Snoussi *et al.*, 2001 ▸). Thus, the unusual structure reported here helps to expand our general knowledge of RNA conformational space.

## Supplementary Material

PDB reference: dimerized UUCG motif, 6e7l

Diffraction images.: https://doi.org/10.15785/SBGRID/712

## Figures and Tables

**Table 1:** Data collection and structure refinement Values in parentheses are for the highest resolution shell.

Wavelength (Å)	0.9792
Resolution range (Å)	77.86–2.59 (2.71–2.59)
Space group	*P*4_1_2_1_2
Unit-cell parameters (Å)	*a* = *b* = 42.0, *c* = 77.9
Total reflections	58704 (7439)
Unique reflections	2473 (294)
Multiplicity	23.7 (25.3)
Completeness (%)	100 (100)
Mean *I*/σ(*I*)	20.0 (1.3)
Wilson *B* factor (Å^2^)	59
*R*_merge_	0.11 (2.98)
*R*_p.i.m._	0.02 (0.59)
CC_1/2_	1.00 (0.53)
*R*_work_/*R*_free_	0.18/0.21 (0.38/0.36)
No. of atoms	
Total	474
Macromolecules	463
Ligands	0
Water	11
R.m.s.d., bonds (Å)	0.012
R.m.s.d., angles (°)	1.94
Coordinate error (maximum likelihood) (Å)	0.58
Phase error (maximum likelihood) (°)	28.98
Clashscore	1.42
Average *B* factor (Å^2^)	
Overall	64
RNA	64
Solvent	63
